# Dietary Pea Fiber Supplementation Improves Glycemia and Induces Changes in the Composition of Gut Microbiota, Serum Short Chain Fatty Acid Profile and Expression of Mucins in Glucose Intolerant Rats

**DOI:** 10.3390/nu9111236

**Published:** 2017-11-12

**Authors:** Zohre Hashemi, Janelle Fouhse, Hyun Seun Im, Catherine B. Chan, Benjamin P. Willing

**Affiliations:** 1 Department of Agricultural, Food and Nutritional Science, University of Alberta, Edmonton, AB T6G 2P5, Canada; zohre.hashemi@ualberta.ca (Z.H.); fouhse@ualberta.ca (J.F.); angela.hs.im@gmail.com (H.S.I.); cbchan@ualberta.ca (C.B.C.); 2 Department of Physiology, University of Alberta, Edmonton, AB T6G 2H7, Canada

**Keywords:** glucose tolerance, gut microbiome, mucins, pea fiber, short chain fatty acids

## Abstract

Several studies have demonstrated the beneficial impact of dried peas and their components on glucose tolerance; however, the role of gut microbiota as a potential mediator is not fully examined. In this study, we investigated the effect of dietary supplementation with raw and cooked pea seed coats (PSC) on glucose tolerance, microbial composition of the gut, select markers of intestinal barrier function, and short chain fatty acid profile in glucose intolerant rats. Male Sprague Dawley rats were fed high fat diet (HFD) for six weeks to induce glucose intolerance, followed by four weeks of feeding PSC-supplemented diets. Cooked PSC improved glucose tolerance by approximately 30% (*p* < 0.05), and raw and cooked PSC diets reduced insulin response by 53% and 56% respectively (*p* < 0.05 and *p* < 0.01), compared to HFD (containing cellulose as the source of dietary fiber). 16S rRNA gene sequencing on fecal samples showed a significant shift in the overall microbial composition of PSC groups when compared to HFD and low fat diet (LFD) controls. At the family level, PSC increased the abundance of *Lachnospiraceae* and *Prevotellaceae* (*p* < 0.001), and decreased *Porphyromonadaceae* (*p* < 0.01) compared with HFD. This was accompanied by increased mRNA expression of mucin genes Muc1, Muc2, and Muc4 in ileal epithelium (*p* < 0.05). Serum levels of acetate and propionate increased with raw PSC diet (*p* < 0.01). These results indicate that supplementation of HFD with PSC fractions can improve glycemia and may have a protective role against HFD-induced alterations in gut microbiota and mucus layer.

## 1. Introduction

Type 2 diabetes (T2D) is the most common form of diabetes mellitus and accounts for 90–95% of 422 million cases of diabetes worldwide, highlighting the importance of prevention and management of this disease. Dietary modifications are established as a critical component of T2D and prediabetes management. Incorporation of healthy foods, such as pulses, with beneficial impacts on glycemic control has been recommended by Diabetes Canada guidelines [1]. Dried peas (*Pisum sativum*) are one of the most common variety of pulses characterized as a rich source of dietary fiber. Peas and their components have been largely studied in terms of their favorable effects on glycemic control and insulin resistance as seen in prediabetes and T2D [2,3,4,5,6]. Most of these beneficial effects have been attributed to pea’s fermentable dietary fiber content and the consequent production of short chain fatty acids (SCFA). In addition, there are indications that pea-derived fiber has microbial modifying properties. In healthy Wistar rats, peas have been shown to possess the strongest bifidogenic properties when compared to other types of pulses including chickpeas, beans, and lentils [7]. Another study reported decreased *Firmicutes* following consumption of pea flour- and pea fiber fractions in diet-induced obese rats [8].

It is well documented that diet is an important factor contributing to the composition of gut microbiota [9,10]. Microbial modifications caused by dietary fibers with prebiotic properties have been linked with improved intestinal permeability as a marker of intestinal barrier function [11,12]. On the other hand, high fat diet (HFD) feeding has been associated with adverse microbial modifications in the microbiota [13,14] and impaired intestinal barrier function [15,16,17]. In turn, disruption of intestinal barrier function plays a role in the pathogenesis of several diseases including diabetes, obesity, and multiple gastrointestinal disorders [18,19,20,21].

We previously showed that the inclusion of raw pea seed coats (PSC) in the diets of glucose intolerant rats resulted in improved glucose homeostasis; however, we did not examine the effect of cooking, as the relevant form of preparation, on these outcomes [4]. Considering the potential of peas to alter gut microbiota due to their fermentable dietary fiber content, the primary objective of the present study was to elucidate the effects of feeding glucose intolerant rats raw and cooked PSC fractions on glycemia and the microbial composition of the gut. Including both raw and cooked PSC preparations in the study allowed examination of the effect of cooking on the potential results. Our secondary objective was to examine the effect of PSC fractions on select markers of gut barrier function, particularly the abundance of toll-like receptors (TLRs), tight junction and mucin proteins, and SCFA production. We hypothesized that dietary supplementation with PSC fractions would improve glycemia and alter the overall microbial composition of the gut compared to control diets. We also hypothesized that PSC-containing diets would partially reverse the HFD-induced changes in intestinal barrier through normalizing the expression of tight junction proteins ZO-1 and occludin, TLRs and mucin proteins.

## 2. Materials and Methods

### 2.1. Animals, Experimental Diets and Tissue Collection

Male Sprague Dawley rats (*n* = 32) were obtained from the Department of Biology, University of Alberta, at eight weeks of age. During one week of acclimatization, animals were housed 2/cage under controlled conditions of temperature and humidity, on a 12-h light/dark cycle with free access to normal chow and water. In brief, rats were fed HFD for six weeks to induce glucose intolerance. They were then randomly assigned to HFD supplemented with either raw (RP) or cooked (CP) PSC. The two control groups were HFD and low fat diet (LFD) with 10% (*w*/*w*) cellulose as the source of dietary fiber, whereas in the RP and CP groups, cellulose was substituted 1:1 (*w*/*w*) with PSC preparations. The complete composition of the diets is provided in Table 1. Rats were fed the treatment diets for four weeks, and weighed once a week to monitor weight gain. Twenty-four hour food intake was also measured on two separate days throughout the four-week period. Body weight gain and food intake were comparable between groups as reported previously [6]. At the end of the study, rats were anesthetized with ketamine (100 mg/kg) and xylazine (1 mg/kg) and euthanized by exsanguination. Segments of ileum and colon were removed and scrapings were collected along with colonic fecal samples and epididymal fat pad and snap frozen in liquid nitrogen. Serum was obtained from blood collected by cardiac puncture (Figure S1). The animal protocols were approved by the Health Sciences Animal Care and Use Committee at the University of Alberta and conformed to the guidelines of the Canadian Council on Animal Care.

### 2.2. Oral Glucose Tolerance Test 

Oral glucose tolerance test (OGTT) was conducted one week before the end of the study following an overnight fast. Fasting blood glucose was measured, and blood was collected for insulin determination. Rats then received an oral glucose gavage of 1 g/kg body weight and blood glucose values obtained at 10, 20, 30, 60, and 120 min using an Accu-Chek Compact Plus glucometer (Roche Diagnostics, Basel, Switzerland). Blood was collected at each time point during OGTT and centrifuged to obtain plasma for insulin analysis with ELISA (Alpco, Salem, NH, USA).

### 2.3. DNA Extraction and Microbial Profiling

Stool pellets from animals were collected for microbial composition analysis at the time of tissue collection. Total DNA was extracted according to manufacturer’s instructions (QIAamp DNA Stool Mini kit, Qiagen, Valencia, CA, USA) with the addition of a 60 s homogenization step (FastPrep instrument, MP Biomedicals, Solon, OH, USA). The V1–V3 region of the 16S rRNA gene fragments was amplified using a set of 33 nucleotide-bar-coded primer pairs (27F; 5′-AGAGTTTGATCMTGGCTCAG-3′, 519R; 5′-GWATTACCGCGGCKGCTG-3′) in triplicate. PCR products were then gel-purified with a QIAquick gel extraction kit (Qiagen, Valencia, CA, USA). The resultant PCR amplicons (100 ng each) were pooled and pyrosequenced with a 454 Titanium platform (Roche, Branford, CT, USA). Specific primers were used to characterize 16S rRNA gene copy numbers of *Bifidobacterium* spp. and total bacteria as previously described [22].

#### Bioinformatics

Sequences were processed using MOTHUR according to the standard operating procedure, accessed on 10 July 2013 [23]. Quality sequences were obtained by removing sequences with ambiguous bases or quality read length less than 200 bases and chimeras identified using chimera.uchime. Quality sequences were aligned to the silva bacterial reference alignment, and operational taxonomic units were generated using a dissimilarity cutoff of 0.03. Sequences were classified using the classify. seqs command with Ribosomal Database Project as reference. Inverse Simpson’s diversity index was used to calculate diversity. Differences in microbial communities between groups were investigated using the phylogeny-based weighted UniFrac distance metric. Significant differences in community structure were determined by analysis of similarity (ANOSIM) in PAST v3.14 [24]. Diversity, similarity, and abundance of bacterial taxa were compared using nonparametric Wilcoxon rank sum test with npar1way, and pairwise multiple comparison analysis was achieved using Dwass, Steel, Critchlow-Flingner multiple comparison procedure (SAS Studio v9.4, SAS Institute Inc., Cary, NC, USA).

### 2.4. RNA Extraction and Real-Time Polymerase Chain Reaction

Total RNA was extracted from ileal and colonic scrapings and frozen epididymal fat using Trizol reagent (Invitrogen, Carlsbad, CA, USA) followed by column-based purification with an RNeasy mini kit (Qiagen, Valencia, CA, USA) according to the manufacturer’s instructions. Reverse transcription was performed on 1 μg of total RNA using a cloned AMV first-strand cDNA synthesis kit (Invitrogen, Carlsbad, CA, USA). Primers used for cDNA amplification by real-time PCR are listed in Table S1. Glyceraldehyde-3-phosphate dehydrogenase (GAPDH) was used as the housekeeping gene for normalization of the target genes expression. PCR reactions were performed using Perfecta SYBR green supermix (Quanta BioSciences, Gaithersburg, MD, USA). All assays were run in duplicate on a ViiAtm 7 PCR cycler (Applied Biosystems, Grand Island, NY, USA).

### 2.5. SCFA Measurement

SCFA concentrations in feces and blood serum were analyzed by gas chromatography. Briefly, approximately 100 mg of feces was diluted with 25% phosphoric acid (1:4 *w*:*v*) mixed thoroughly and centrifuged. Supernatant was mixed with an internal standard (24.5 mml/L isocaproic acid). Samples were measured by injection into a Stabilwax-DA column (30 m × 0.53 mm i.d. × 0.5 μm film thickness; Restek Corporation, Bellefonte, PA, USA) on a Varian gas chromatograph (Model 3800; Varian Analytical Instruments, Palo Alto, CA, USA) using an autosampler (Model 8400; Varian Inc., Walnut Creek, CA, USA). Final results were normalized by the weight of each sample used.

### 2.6. Statistical Analysis

Statistical analyses were conducted using GraphPad Prism 6 (Graphpad Software Inc., La Jolla, CA, USA). Prior to analyses, data were tested for normality of distribution by the Shapiro-Wilk test. For gene expression data, 2-ΔΔCT analysis was used. Statistically significant differences were determined by two-way repeated measures analysis of variance (ANOVA), one-way ANOVA for parametric and Kruskal-Wallis test for nonparametric data. Bonferroni’s and Dunn’s post-hoc comparison tests, corrected for multiple comparisons, were performed as appropriate to assess differences between individual diet groups. Incremental area under the curve (IAUC) was calculated for glucose and insulin. All data are expressed as means ± SEM and a *p*-value < 0.05 was considered to be significant.

## 3. Results

### 3.1. Oral Glucose Tolerance Test and Insulin Measurement

**Figure 1:**
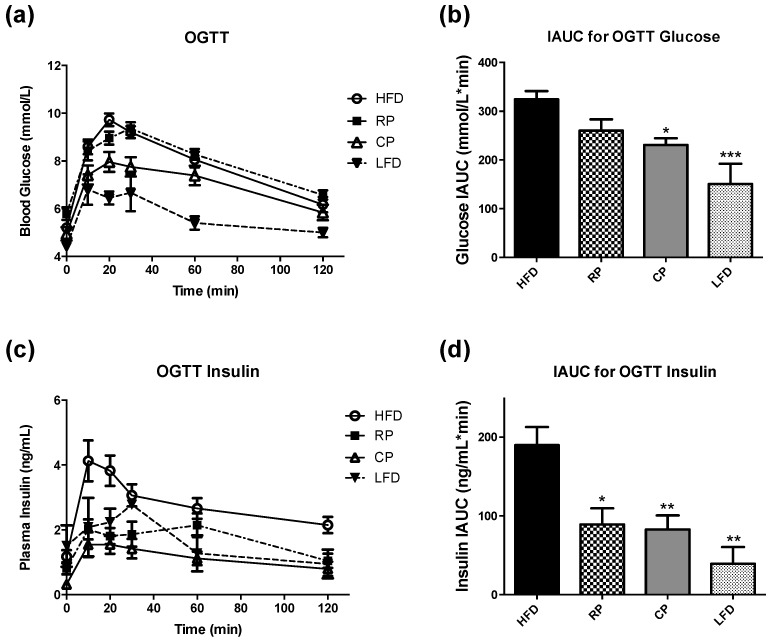
Effect of 4 weeks of feeding a HFD supplemented with raw or cooked PSC on oral glucose tolerance test (OGTT) and the corresponding insulin response. (**a**) Concentrations of blood glucose measured basally and following oral administration of 1 g/kg glucose; (**b**) IAUC calculated for glucose levels during OGTT; (**c**) Plasma insulin levels during OGTT; (**d**) incremental area under the curve (IAUC) for insulin during OGTT. The data represent mean ± SEM, *n* = 4–14. Significant differences seen at different time points of OGTT are explained in the text, and differences between IAUCs are shown here. * *p* < 0.05, ** *p* < 0.01 and *** *p* < 0.001 show significant difference compared with HFD. Reproduced from Hashemi et al. [6] with permission.

OGTT results are shown as glucose levels over the course of the 120-min test and IAUC values of blood glucose levels (Figure 1a,b). The LFD group had decreased glucose concentrations at time 10 (*p* < 0.05), 20, 30 and 60 min (*p* < 0.001) compared with HFD. CP showed significantly lower glucose response at time 10 (*p* < 0.05), 20 (*p* < 0.001) and 30 min (*p* < 0.01) compared to HFD. Both LFD and CP had lower IAUC values when compared to the HFD group (*p* < 0.001 and *p* < 0.05, respectively). Plasma insulin levels measured during OGTT and their corresponding IAUC are shown in Figure 1c,d. The LFD group had lower plasma insulin concentrations than the HFD group at time 10 min (*p* < 0.01). Compared to the HFD, RP rats showed decreased insulin response at time 10 and 20 min (*p* < 0.001); CP group had lower plasma insulin concentrations at time 10 (*p* < 0.001), 20 (*p* < 0.001), 30 (*p* < 0.01), 60 (*p* < 0.01), and 120 min (*p* < 0.05). IAUC for insulin response during OGTT showed that RP, CP, and LFD had smaller IAUC values compared to HFD (*p* < 0.05, *p* < 0.01, and *p* < 0.01, respectively).

### 3.2. Microbial Community Structure

**Figure 2:**
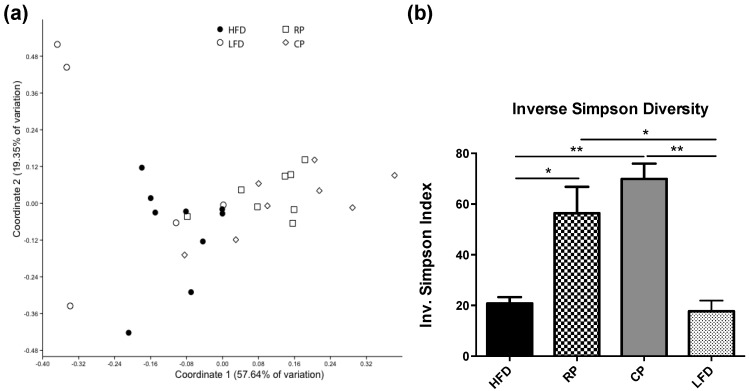
Effect of feeding PSC-supplemented diets on fecal microbial composition. (**a**) Fecal bacterial communities clustered using PCoA analysis of weighted UniFrac distances, analyzed by ANOSIM. The percentage of variation explained by each coordinate is shown in parentheses. HFD and LFD clustered together (*p* > 0.05) and cooked (CP) and raw (RP) clustered together (*p* > 0.05); however, HFD and LFD clustered separately from CP and RP (*p* < 0.05); (**b**) Inverse Simpson diversity index as a measure of diversity within each sample. Inverse Simpson diversity indices differed significantly among the groups (*p* < 0.001). Bars are means ± SEM, *n* = 6–8, ** *p* < 0.01, * *p* < 0.05.

After quality filtering, a mean of 1275 ± 36 sequences were obtained per sample. Sequences were subsampled to the sample with the lowest number of reads (794). Addition of RP or CP to the HFD induced a substantial shift in the composition and structure of the fecal microbial community. Grouping of microbial composition by dietary treatment is reflected in the principal coordinate analysis (PCoA) plot in Figure 2a. The CP and RP treatments distinctly clustered separately from both HFD and LFD treatment groups (ANOSIM, *p* < 0.05), whereas the two PSC treatments did not differ from each other (*p* = 0.179). There was an increase in diversity in response to both RP and CP fractions versus HFD and LFD as indicated by inverse Simpson diversity index (Figure 2b, *p* < 0.05 and *p* < 0.01 respectively).

At the phylum level, there was an overall decrease in the proportion of *Bacteroidetes* (*p* < 0.05) in CP as compared to HFD (Table 2). Similar overall patterns were seen with RP, however they did not reach statistical significance. *Firmicutes* abundance was not different between groups (*p* = 0.06).

The effect of dietary treatments on fecal microbial composition at the family level is also described in Table 2. The effects of PSC for the most part did not return the microbial population to that seen in LFD rats. The one exception to that was the population of *Porphyromonadaceae*. This is the only bacterial family that was affected by PSC the same as LFD, with all three groups showing significantly lower abundance compared to HFD (*p* < 0.05). *Acidaminococcaceae*, on the other hand, was significantly decreased in HFD compared to LFD (*p* < 0.05); however, this family was not detected in either of the PSC groups. The separation of PSC groups by multivariate analysis was largely associated with the relative proportion of bacteria from the *Lachnospiraceae* family. *Lachnospiraceae* was also the most abundant bacterial family in all treatment groups. There was also an increase in *Prevotellaceae* in RP and CP groups. The pattern of alterations in bacterial populations at all taxonomic levels was very consistent between the two PSC groups, however the CP had a slightly stronger effect on microbial populations, shifting further away from HFD microbiota. Relative abundance of *Bifidobacterium*, as indicated by qPCR, was low and not different between treatment groups.

### 3.3. Gene Expression of TLR, Tight Junction Proteins, and Mucins

**Figure 3:**
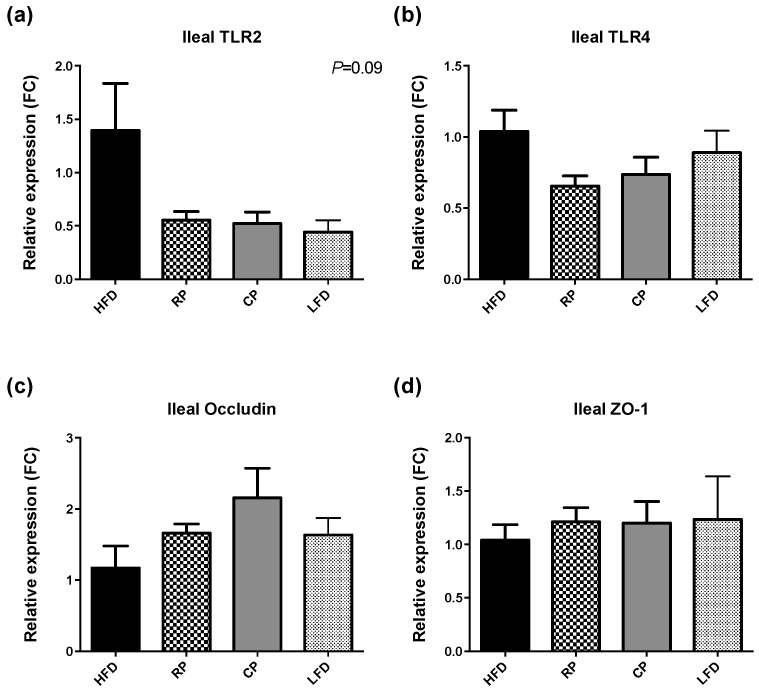
mRNA expression of TLRs and tight Junction proteins. Mean relative mRNA expression (FC, fold change) of (**a**) TLR2; (**b**) TLR4; (**c**) occludin and (**d**) ZO-1 in ileum of the rats normalized to glyceraldehyde-3-phosphate dehydrogenase (GAPDH) expression. Data are means ± SEM, *n* = 5–8. No significant differences were found between groups.

Following four weeks of feeding experimental diets, ileal expression of TLR2 showed a pattern of elevated expression in HFD relative to other treatments (Figure 3a, *p* = 0.09). TLR4 expression did not show the same trend (Figure 3b, *p* = 0.19). Relative mRNA expression of occludin and ZO-1 in the ileum was numerically highest in CP but did not achieve significance (Figure 3c,d, *p* = 0.16 and *p* = 0.86 respectively).

**Figure 4:**
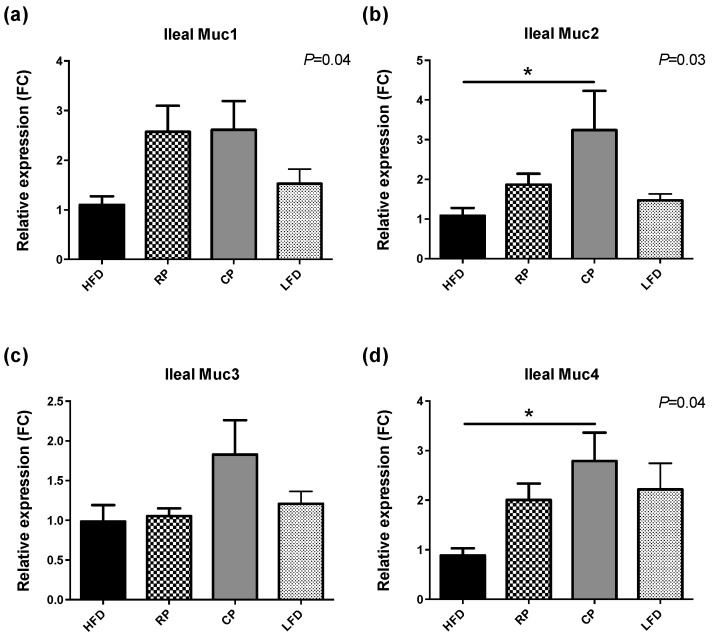
mRNA expression of mucins. Relative mRNA expression (FC, fold change) of (**a**) Muc1; (**b**) Muc2; (**c**) Muc3 and (**d**) Muc4 in the ileum. Gene expression data was normalized to GAPDH as the house-keeping gene and presented as means ± SEM, *n* = 6–8. There was a significant effect of treatment for relative expression of Muc1, Muc2, and Muc4 (*p* < 0.05). CP group showed increased expression of Muc2 and Muc4 genes when compared to HFD group (* *p* < 0.05).

Relative expressions of mucin genes (Muc1, Muc2, Muc3 and Muc4) in the ileum are shown in Figure 4. After four weeks of PSC supplementation, Muc1, Muc2, and Muc4 mRNA expression levels differed between the diet groups (*p* < 0.05). In particular, CP rats showed elevated expression of Muc2 and Muc4 mRNA compared to the HFD group (*p* < 0.05). No significant differences were observed in the ileal expression of Muc3 (Figure 4c).

### 3.4. SCFA in Serum and Fecal Samples

**Figure 5:**
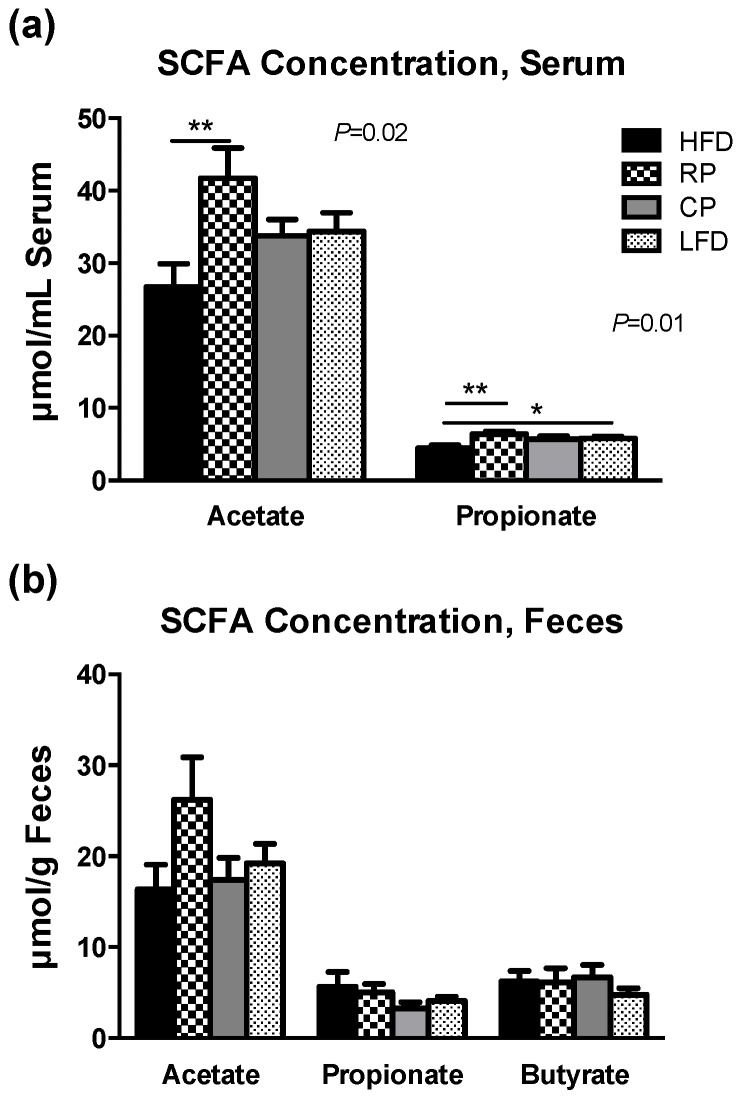
SCFA concentration (μmol/g) in (**a**) serum and (**b**) feces of rats following four weeks of PSC-supplemented or control diets. Data are presented as means ± SEM, *n* = 6–8. Serum acetate and propionate concentrations were significantly different between the groups. Asterisks show significant difference compared to HFD (** *p* < 0.01, * *p* < 0.05). Fecal SCFAs concentrations were not statistically different between groups.

Serum levels of SCFA acetate and propionate varied significantly between the groups (Figure 5a, *p* < 0.05 and *p* < 0.01, respectively); RP had significantly higher levels of acetate and propionate compared to HFD, while LFD showed a significant increase in propionate. Fecal concentrations of acetate, propionate, and butyrate were comparable between the groups (Figure 5b).

## 4. Discussion

The present study evaluated the effects of PSC feeding on glycemia, the composition of gut microbiota, select markers of intestinal barrier function, and SCFA profile in HFD-induced glucose intolerant rats. We found that adding PSC to the diet of glucose intolerant rats had a similar effect on overall microbial composition whether it was cooked or not. Cooked PSC fractions were, however, the only pea group to improve glycemia. PSC feeding also increased serum SCFA concentrations and ileal mucin gene expression but did not have a significant effect on tight junction proteins.

PSC supplementation to HFD-induced glucose-intolerant rats resulted in improved glycemia as measured by OGTT. This effect appeared to be dependent on the method of preparation since raw PSC fractions were not as beneficial as cooked ones. Analysis of PSC fractions showed that both preparations were composed of similar proportions of soluble and insoluble dietary fiber [6], which means these results could not be explained by the effect of cooking on dietary fiber composition, as documented before [25]. We speculate this resulted from separation and/or hydration of the fiber components along with partial solubilization and depolymerization of select dietary fiber substances upon cooking [26,27], and its consequent impact on the interactions between dietary fiber and gut microbiota. Consistent with improved glucose tolerance, we showed insulin secretion in response to glucose ingestion was lower in both PSC-supplemented diets. CP also led to reduced fasting insulin. In other words, PSC-fed rats required less insulin to maintain glucose at levels lower than those of HFD-fed rats.

Both RP and CP increased the abundance of *Lachnospiraceae*, a butyrate-producing family that belongs to the phylum *Firmicutes* [28]. This finding is consistent with a report of increased relative abundance of *Lachnospiraceae* in pigs fed resistant starch [29]. While we did not detect increased butyrate concentrations in feces, a previous study by our group indicated an increase in gut-derived 3-hydroxybutyrate in the serum with RP inclusion in the diet [30]. Considering the energy substrate role of butyrate and its fast absorption by colonocytes [31,32], it is possible that fecal butyrate did not reflect its production in the gut. In addition, there was an increase in *Prevotellaceae* in both PSC groups, suggesting reactivity of this family to fiber type, as the results are similar to previous animal studies showing that diets enriched in pectin [33] or whole grains such as oats [34] increased the abundance of *Prevotellaceae* family. Furthermore, African children, who consumed diets high in dietary fiber, were found to harbor a large population of the genus Prevotella, one of the four genera belonging to the family *Prevotellaceae*, compared to European children, who lacked this bacterium and consumed a typical low-fiber western diet [35]. Likewise, Wu et al. [36] reported that in adults, dietary fiber intake was associated with a microbiota dominated by Prevotella. Prevotella species contain bacterial genes that enable them to utilize polysaccharides such as water-soluble xylans [37]. This is in keeping with our previously reported PSC fiber analysis showing that xylose, the primary building block for xylan, is present in both raw and cooked PSC preparations [6]. In another study, hamsters receiving a diet supplemented with pineapple-derived fiber (containing xylan and xyloglucan in the form of hemicellulose) exhibited altered gut microbiota when compared to cellulose [38]. Similar to our results, this study suggests that not all sources of insoluble dietary fiber have the ability to favourably change gut microbiota and some, such as those composed of xylans, could display stronger beneficial properties.

Previous studies examining the impact of peas or pea-derived components on gut microbiota have reported increased *Bifidobacterium* population [7,39], increased Lactobacillus counts [39], reductions in the abundance of *Firmicutes* [8], and substantial changes in the structure of this phylum [3]. However, these studies were performed under considerably different conditions than ours. Increased *Bifidobacterium*, for instance, was found following consumption of whole peas supplemented to a balanced diet based on AIN-93G diet. Additionally, those experimental diets were not matched for total dietary fiber and pea diet contained more than double the amount of dietary fiber in the control group [7]. Eslinger et al. [8] used commercially available yellow pea–derived components (fiber, starch, and flour) as a part of basal AIN-93M diet with a slightly higher proportion of total dietary fiber (13% *wt*/*wt*). The five-week duration of their study was also longer than ours. Changes in the composition of *Firmicutes* were reported in hamsters fed untreated commercially available pea flours from whole seeds and seed coats; differences were compared to a control diet containing a lower amount of dietary fiber [3].

At the phylum level, HFD-induced metabolic disorders including obesity and insulin resistance are sometimes characterized by an increased ratio of *Firmicutes* to *Bacteroidetes* [13,40,41,42]. However, several other studies do not support this link [43,44,45,46]. In our study, we observed no difference in the abundance of *Firmicutes* and a significant reduction in the proportion of *Bacteroidetes* in CP compared to HFD, whereas RP displayed a similar non-significant pattern.

We also examined the effect of PSC diets on the expression of TLR2 and TLR4 in the ileum. TLR4 is essential for the recognition of lipopolysaccharide (LPS) originating from the outer membrane of Gram-negative bacteria, and TLR2 is implicated in the recognition of Gram-positive bacterial components such as peptidoglycan (PGN) [47,48]. HFD feeding has been shown to result in increased expression of TLR2 in adipocytes of insulin resistant mice [49]. We observed a trend toward a reduced expression level of TLR2 in ileal tissue in both RP and CP compared to HFD, which was not significant. TLR2 gene expression in adipose tissue and TLR2 and TLR4 gene expression in colonic epithelium were also examined, and no differences were observed between the groups (Figure S2).

SCFA acetate and propionate were significantly different between the treatment groups with RP having the highest concentrations. SCFA play an important role in energy homeostasis and suppression of inflammatory processes through activation of G-protein coupled receptors 43 and 41 [50]. Similar to our findings, dietary pectin resulted in increased levels of acetate and propionate in HFD-fed obese rats [51]. Propionate is known to stimulate glucagon-like peptide-1 (GLP-1) release in rats and human [52], which is consistent with our previous finding of increased GLP-1 secretion in PSC fed rats [6]. It has also been shown that propionate increases Muc2 expression in vitro [53], which can in turn affect epithelial integrity.

Expression of mucin genes in the ileum was significantly influenced by CP, which had higher expression of Muc2 and Muc4 compared to HFD. This finding supports our hypothesis that PSC support the intestinal barrier because mucins are important for the integrity of the mucus layer. In mice, HFD feeding is associated with changes in the oligosaccharide chains of mucins and consequently their altered composition [54] and HFD decreases expression of duodenal Muc2 [55]. Muc2 is the main structural component of mucus layer, and hence important for the protective function of this layer [56]. In addition, emerging evidence on the role of mucin-degrading bacteria *Akkermansia muciniphila* in gut barrier function further proves the importance of mucins for the homeostatic actions of mucus layer. It has been shown that the population of these bacteria was decreased following HFD feeding in mice; and was restored by administration of oligofructose, which was accompanied by an increase in the mucus layer thickness [57]. Our results suggest that PSC-containing diets, especially when cooked, could reverse the effect of HFD on mucin expression and potentially benefit intestinal barrier integrity, which was not directly measured. Expression of mucin genes Muc1 and Muc2 was also measured in the colon. However, no significant differences were found (data not shown). Relative expression of tight junction proteins occludin and ZO-1 was assessed in the ileum, but was not different between any treatment group. It is important to keep in mind that gene expression data is not an optimal predictor for the functions of tight junction proteins since their organization and distribution plays a more important role in their functionality.

There are a few limitations that should be kept in mind when interpreting the findings of this study. The specific microbiota composition derived from gene pyrosequencing is highly affected by the 16S rRNA regions and primers chosen for amplification [58,59]. We also recognize that gene expression analysis of tight junction proteins is not conclusive without considering the importance of their structure and distribution, which could be modified independently from the abundance of their proteins.

## 5. Conclusions

The present study demonstrates that inclusion of raw and cooked PSC fractions in diets of glucose intolerant rats alters the composition of gut microbiota, while CP also improves glucose tolerance. This observation was accompanied by an increased expression of mRNA encoding mucin proteins in the ileum and a rise in serum concentrations of acetate and propionate. These effects, both on microbiota composition and SCFA profile and protective gene expression, were consistently stronger in the CP group. Our findings suggest a potential protective role for PSC fractions against HFD-induced alterations in the microbial composition of the gut and elements of gut barrier function.

## Figures and Tables

**Table 1:** Composition of the experimental diets (g/kg).

	HFD	HFD + PSC	LFD
Canola sterine	99.5	99.5	29.85
Flaxseed oil	6	6	1.8
Sunflower oil	94.5	94.5	28.35
Casein	270	263	270
Dextrose	189	189	255
Corn starch	169	169	245
Cellulose	100	0	100
Pea seed coat (dwt) ^1^	0	107	0
L-methionine	2.5	2.5	2.5
Mineral mix	51	51	51
Vitamin mix	10	10	7.6
Inositol	6.3	6.3	6.3
Choline chloride	2.8	2.8	2.8
Carbohydrate (% dwt)	36	36	51
Fat (% dwt)	20	20	6
Protein (% dwt)	27	27	27
Fiber (% dwt)	10	10	10

^1^ dwt, dry weight. Pea seed coats (PSC) composition has been described previously in detail [6]. The amount of PSC was adjusted to provide 100 g fiber per kg diet to match the amount of cellulose in high fat diet (HFD) and low fat diet (LFD). The decrease in the amount of casein in the PSC diets reflects the amount of protein contributed by the PSC.

**Table 2:** Relative abundance of bacteria at phylum and family levels in feces.

Title 1	HFD	RP	CP	LFD	SEM	*p* Value
**Phyla**						
*Firmicutes*	63.9	71.3	77.4	65.6	2.01	0.06
*Bacteroidetes*	34.2	25.4	19.5 *	29.6	1.62	0.023
*Proteobacteria*	0.49	0.36	0.49	2.43	0.14	0.09
*TM7*	0.17	0.77 *	0.65	0.34	0.19	0.024
*Tenericutes*	0.11	0.57	0.35	0.31	0.31	0.36
*Deferribacteres*	0.17	0.11	0.13	0.02	0.04	0.39
*Actinobacteria*	0.05	0.00	0.05	0.04	0.03	0.34
*Verrucomicrobia*	0.00	0.00	0.02	0.04	0.03	0.15
**Family**						
*Lachnospiraceae*	34.6	51.0 *^,†^	55.0 *^,†^	24.8	2.72	<0.001
*Bacteroidaceae*	12.88	10.41	6.61	17.53	2.21	0.06
*Ruminococcaceae*	19.63	11.92	12.72	16.50	1.42	0.46
*Porphyromonadaceae*	13.4	5.45 *	4.10 *	3.8 *	1.49	0.01
*Clostridiales* ^1^	3.53	5.48	7.01 *	4.70	0.60	0.02
*Bacteroidales* ^1^	4.44	5.51	5.09	4.13	0.58	0.58
*Erysipelotrichaceae*	0.39 ^†^	0.3 ^†^	0.17 ^†^	12.8	0.09	0.001
*Acidaminococcaceae*	0.60	0.00	0.00	3.17 *	1.64	0.001
*Lactobacillaceae*	2.33	1.31	1.16	1.13	0.50	0.98
*Peptostreptococcaceae*	2.16	1.24	0.38	0.31	0.50	0.15
*Firmicutes* ^2^	0.58 ^†^	0.36 ^†^	0.50 ^†^	1.55	0.08	0.011
*Sutterellaceae*	0.20	0.08	0.17	0.48	0.04	0.28
*Prevotellaceae*	0.11	1.23 *^,†^	0.69 *^,†^	0.02	0.39	<0.001
*TM7_family_incertae_sedis*	0.24	1.23	0.54	0.34	0.12	0.16
*Verrucomicrobiaceae*	0.00	0.00	0.03	0.02	0.07	0.27
*Pasteurellaceae*	0.06	0.14	0.02	1.39	0.06	0.08
*Rikenellaceae*	0.16	0.17	0.02	0.06	0.32	0.14
*Anaeroplasmataceae*	0.14	0.63	0.41	0.21	0.02	0.50
*Proteobacteria* ^2^	0.08	0.03	0.08	0.00	0.06	0.61
*Peptostreptococcaceae*	0.09	0.17	0.22	0.15	0.05	0.73
*Deferribacteraceae*	0.20	0.09	0.08	0.06	0.02	0.60
*Streptococcaceae*	0.05	0.08	0.09	0.27	0.02	0.11
*Clostrideaceae*	0.05	0.02	0.02	0.38	0.03	0.66
*Enteriobacteriaceae*	0.08	0.06	0.03	0.15	0.04	0.66
*Clostridiales_Incertae_Sedis_XIII*	0.08	0.00	0.05	0.10	0.04	0.06
*Desulfovibrionaceae*	0.02	0.09	0.03	0.10	0.06	0.37
*Coriobacteriaceae*	0.02	0.00	0.02	0.08	0.04	0.08
*Bifidobacteria* ^3^	0.123	0.004	0.003	0.003	0.09	0.72

^1^ Unclassified Family or Order; ^2^ Unclassified Order and Family of Phylum; ^3^ As a percentage of total bacteria as determined by qPCR; * Significant difference compared with HFD (*p* < 0.05); ^†^ Significant difference compared with LFD (*p* < 0.05); *n* = 6–8.

## Supplementary Materials

The following are available online at http://www.mdpi.com/2072-6643/9/11/1236/s1, Supplementary Table S1. Primer sequences for RT-PCR; Supplementary Figure S1. Schematic diagram showing the experimental outline of the study; Supplementary Figure S2. mRNA expression of TLRs in adipose tissue and colon.

## References

[1] Canadian Diabetes Association Clinical Practice Guidelines Expert Committee (2013). Canadian diabetes association 2013 clinical practice guidelines for the prevention and management of diabetes in Canada. Can. J. Diabetes.

[2] Dahl W.J., Foster L.M., Tyler R.T. (2012). Review of the health benefits of peas (*Pisum sativum* L.). Br. J. Nutr..

[3] Marinangeli C.P., Krause D., Harding S.V., Rideout T.C., Zhu F., Jones P.J.H. (2011). Whole and fractionated yellow pea flours modulate insulin, glucose, oxygen consumption, and the caecal microbiome in Golden Syrian hamsters. Appl. Physiol. Nutr. Metab..

[4] Whitlock K.A., Kozicky L., Jin A., Yee H., Ha C., Morris J., Field C.J., Bell R.C., Ozga J.A., Chan C.B. (2012). Assessment of the mechanisms exerting glucose-lowering effects of dried peas in glucose-intolerant rats. Br. J. Nutr..

[5] Mollard R.C., Luhovyy B.L., Smith C., Anderson G.H. (2014). Acute effects of pea protein and hull fibre alone and combined on blood glucose, appetite, and food intake in healthy young men—A randomized crossover trial. Appl. Physiol. Nutr. Metab..

[6] Hashemi Z., Yang K., Yang H., Jin A., Ozga J., Chan C.B. (2015). Cooking enhances beneficial effects of pea seed coat consumption on glucose tolerance, incretin, and pancreatic hormones in high-fat-diet-fed rats. Appl. Physiol. Nutr. Metab..

[7] Da S Queiroz-Monici K., Costa G.E., da Silva N., Reis S.M., de Oliveira A.C. (2005). Bifidogenic effect of dietary fiber and resistant starch from leguminous on the intestinal microbiota of rats. Nutrition.

[8] Eslinger A.J., Eller L.K., Reimer R.A. (2014). Yellow pea fiber improves glycemia and reduces Clostridium leptum in diet-induced obese rats. Nutr. Res..

[9] Turnbaugh P.J., Hamady M., Yatsunenko T., Cantarel B.L., Duncan A., Ley R.E., Sogin M.L., Jones W.J., Roe B.A., Affourtit J.P. (2009). A core gut microbiome in obese and lean twins. Nature.

[10] Voreades N., Kozil A., Weir T.L. (2014). Diet and the development of the human intestinal microbiome. Front. Microbiol..

[11] Cani P.D., Possemiers S., Van de Wiele T., Guiot Y., Everard A., Rottier O., Geurts L., Naslain D., Neyrinck A., Lambert D.M. (2009). Changes in gut microbiota control inflammation in obese mice through a mechanism involving GLP-2-driven improvement of gut permeability. Gut.

[12] Neyrinck A.M., Van Hee V.F., Piront N., De Backer F., Toussaint O., Cani P.D., Delzenne N.M. (2012). Wheat-derived arabinoxylan oligosaccharides with prebiotic effect increase satietogenic gut peptides and reduce metabolic endotoxemia in diet-induced obese mice. Nutr. Diabetes.

[13] Turnbaugh P.J., Ley R.E., Mahowald M.A., Magrini V., Mardis E.R., Gordon J.I. (2006). An obesity-associated gut microbiome with increased capacity for energy harvest. Nature.

[14] Cani P.D., Amar J., Iglesias M.A., Poggi M., Knauf C., Bastelica D., Neyrinck A.M., Fava F., Tuohy K.M., Chabo C. (2007). Metabolic endotoxemia initiates obesity and insulin resistance. Diabetes.

[15] Cani P.D., Bibiloni R., Knauf C., Waget A., Neyrinck A.M., Delzenne N.M., Burcelin R. (2008). Changes in gut microbiota control metabolic endotoxemia-induced inflammation in high-fat diet-induced obesity and diabetes in mice. Diabetes.

[16] De La Serre C.B., Ellis C.L., Lee J., Hartman A.L., Rutledge J.C., Raybould H.E. (2010). Propensity to high-fat diet-induced obesity in rats is associated with changes in the gut microbiota and gut inflammation. Am. J. Physiol. Gastrointest. Liver Physiol..

[17] Lam Y.Y., Ha C.W., Campbell C.R., Mitchell A.J., Dinudom A., Oscarsson J., Cook D.I., Hunt N.H., Caterson I.D., Holmes A.J. (2012). Increased gut permeability and microbiota change associate with mesenteric fat inflammation and metabolic dysfunction in diet-induced obese mice. PLoS ONE.

[18] Vaarala O., Atkinson M.A., Neu J. (2008). The “perfect storm” for type 1 diabetes: The complex interplay between intestinal microbiota, gut permeability, and mucosal immunity. Diabetes.

[19] Moreira A.P., Texeira T.F., Ferreira A.B., Peluzio Mdo C., Alfenas Rde C. (2012). Influence of a high-fat diet on gut microbiota, intestinal permeability and metabolic endotoxaemia. Br. J. Nutr..

[20] Lee H., Lee I.S., Choue R. (2013). Obesity, inflammation and diet. Pediatr. Gastroenterol. Hepatol. Nutr..

[21] Geurts L., Neyrinck A.M., Delzenne N.M., Knauf C., Cani P.D. (2014). Gut microbiota controls adipose tissue expansion, gut barrier and glucose metabolism: Novel insights into molecular targets and interventions using prebiotics. Benef. Microbes.

[22] Rinttila T., Kassinen A., Malinen E., Krogius L., Palva A. (2004). Development of an extensive set of 16S rDNA-targeted primers for quantification of pathogenic and indigenous bacteria in faecal samples by real-time PCR. J. Appl. Microbiol..

[23] Schloss P.D., Gevers D., Westcott S.L. (2011). Reducing the effects of PCR amplification and sequencing artifacts on 16S rRNA-based studies. PLoS ONE.

[24] Hammer Ø., Harper D.A.T., Ryan P.D. (2001). PAST: Paleontologial statistics software package for education. Palaeontol. Electron..

[25] Vasishtha H., Srivastava R.P. (2013). Effect of soaking and cooking on dietary fibre components of different type of chickpea genotypes. J. Food Sci. Technol..

[26] Harding S.V., Sapirstein H.D., Rideout T.C., Marinangeli C.P., Dona A.K., Jones P.J. (2014). Consumption of wheat bran modified by autoclaving reduces fat mass in hamsters. Eur. J. Nutr..

[27] Marconi E., Ruggeri S., Cappelloni M., Leonardi D., Carnovale E. (2000). Physicochemical, nutritional, and microstructural characteristics of chickpeas (*Cicer arietinum* L.) and common beans (*Phaseolus vulgaris* L.) following microwave cooking. J. Agric. Food Chem..

[28] Vital M., Howe A.C., Tiedje J.M. (2014). Revealing the bacterial butyrate synthesis pathways by analyzing (meta)genomic data. MBio.

[29] Umu O.C., Frank J.A., Fangel J.U., Oostindjer M., da Silva C.S., Bolhuis E.J., Bosch G., Willats W.G.T., Pope P.B., Diep D.B. (2015). Resistant starch diet induces change in the swine microbiome and a predominance of beneficial bacterial populations. Microbiome.

[30] Chan C.B., Gupta J., Kozicky L., Hashemi Z., Yang K. (2014). Improved glucose tolerance in insulin-resistant rats after pea hull feeding is associated with changes in lipid metabolism-targeted transcriptome. Appl. Physiol. Nutr. Metab..

[31] Bergman E.N. (1990). Energy contributions of volatile fatty acids from the gastrointestinal tract in various species. Physiol. Rev..

[32] Boets E., Deroover L., Houben E., Vermeulen K., Gomand S.V., Delcour J.A., Verbeke K. (2015). Quantification of in Vivo Colonic Short Chain Fatty Acid Production from Inulin. Nutrients.

[33] Ivarsson E., Roos S., Liu H.Y., Lindberg J.E. (2014). Fermentable non-starch polysaccharides increases the abundance of Bacteroides-Prevotella-Porphyromonas in ileal microbial community of growing pigs. Animal.

[34] Zhou A.L., Hergert N., Rompato G., Lefevre M. (2015). Whole grain oats improve insulin sensitivity and plasma cholesterol profile and modify gut microbiota composition in C57BL/6J mice. J. Nutr..

[35] De Filippo C., Cavalieri D., Di Paola M., Ramazzotti M., Poullet J.B., Massart S., Collini S., Pieraccini G., Lionetti P. (2010). Impact of diet in shaping gut microbiota revealed by a comparative study in children from Europe and rural Africa. Proc. Natl. Acad. Sci. USA.

[36] Wu G.D., Chen J., Hoffmann C., Bittinger K., Chen Y.Y., Keilbaugh S.A., Bewtra M., Knights D., Walters W.A., Knight R. (2011). Linking long-term dietary patterns with gut microbial enterotypes. Science.

[37] Flint H.J., Scott K.P., Duncan S.H., Louis P., Forano E. (2012). Microbial degradation of complex carbohydrates in the gut. Gut Microbes.

[38] Huang Y.L., Tsai Y.H., Chow C.J. (2014). Water-insoluble fiber-rich fraction from pineapple peel improves intestinal function in hamsters: Evidence from cecal and fecal indicators. Nutr. Res..

[39] Chen H., Mao X., He J., Yu B., Huang Z., Yu J., Zheng P., Chen D. (2013). Dietary fibre affects intestinal mucosal barrier function and regulates intestinal bacteria in weaning piglets. Br. J. Nutr..

[40] Ley R.E., Turnbaugh P.J., Klein S., Gordon J.I. (2006). Microbial ecology: Human gut microbes associated with obesity. Nature.

[41] Hildebrandt M.A., Hoffmann C., Sherrill-Mix S.A., Keilbaugh S.A., Hamady M., Chen Y.Y., Kinght R., Ahima R.S., Bushman F., Wu G.D. (2009). High-fat diet determines the composition of the murine gut microbiome independently of obesity. Gastroenterology.

[42] Murphy E.F., Cotter P.D., Healy S., Marques T.M., O’Sullivan O., Fouhy F., Clarke S.F., O’Toole P.W., Quigley E.M., Stanton C. (2010). Composition and energy harvesting capacity of the gut microbiota: Relationship to diet, obesity and time in mouse models. Gut.

[43] Zhang H., DiBaise J.K., Zuccolo A., Kudrna D., Braidotti M., Yu Y., Parameswaran P., Crowell M.D., Wing R., Rittmann B.E. (2009). Human gut microbiota in obesity and after gastric bypass. Proc. Natl. Acad. Sci. USA.

[44] Larsen N., Vogensen F.K., van den Berg F.W., Nielsen D.S., Andreasen A.S., Pedersen B.K., Abu AI-Soud W., Sorensen S.J., Hansen L.H., Jakobsen M. (2010). Gut microbiota in human adults with type 2 diabetes differs from non-diabetic adults. PLoS ONE.

[45] Schwiertz A., Taras D., Schafer K., Beijer S., Bos N.A., Donus C., Hardt P.D. (2010). Microbiota and SCFA in lean and overweight healthy subjects. Obesity.

[46] Wu X., Ma C., Han L., Nawaz M., Gao F., Zhang X.Y., Yu P.B., Zhao C.A., Li L.C., Zhou A.P. (2010). Molecular characterisation of the faecal microbiota in patients with type II diabetes. Curr. Microbiol..

[47] McCusker R.H., Kelley K.W. (2013). Immune-neural connections: How the immune system’s response to infectious agents influences behavior. J. Exp. Biol..

[48] Takeuchi O., Akira S. (2001). Toll-like receptors; their physiological role and signal transduction system. Int. Immunopharmacol..

[49] Murakami K., Bujo H., Unoki H., Saito Y. (2007). High fat intake induces a population of adipocytes to co-express TLR2 and TNFalpha in mice with insulin resistance. Biochem. Biophys. Res. Commun..

[50] Maslowski K.M., Vieira A.T., Ng A., Kranich J., Sierro F., Yu D., Schilter H.C., Rolph M.S., Mackay F., Artis D. (2009). Regulation of inflammatory responses by gut microbiota and chemoattractant receptor GPR43. Nature.

[51] Adam C.L., Gratz S.W., Peinado D.I., Thomson L.M., Garden K.E., Williams P.A., Richardson A.J., Ross A.W. (2016). Effects of Dietary Fibre (Pectin) and/or Increased Protein (Casein or Pea) on Satiety, Body Weight, Adiposity and Caecal Fermentation in High Fat Diet-Induced Obese Rats. PLoS ONE.

[52] Spreckley E., Murphy K.G. (2015). The L-Cell in Nutritional Sensing and the Regulation of Appetite. Front. Nutr..

[53] Burger-van Paassen N., Vincent A., Puiman P.J., van der Sluis M., Bouma J., Boehm G., van Goudoever J.B., Van Seuningen I., Renes I.B. (2009). The regulation of intestinal mucin MUC2 expression by short-chain fatty acids: Implications for epithelial protection. Biochem. J..

[54] Mastrodonato M., Mentino D., Portincasa P., Calamita G., Liquori G.E., Ferri D. (2014). High-fat diet alters the oligosaccharide chains of colon mucins in mice. Histochem. Cell Biol..

[55] Schulz M.D., Atay C., Heringer J., Romrig F.K., Schwitalla S., Aydin B., Ziegler P.K., Varga J., Reindl W., Pommerenke C. (2014). High-fat-diet-mediated dysbiosis promotes intestinal carcinogenesis independently of obesity. Nature.

[56] Linden S.K., Florin T.H., McGuckin M.A. (2008). Mucin dynamics in intestinal bacterial infection. PLoS ONE.

[57] Everard A., Belzer C., Geurts L., Ouwerkerk J.P., Druart C., Bindels L.B., Guiot Y., Derrien M., Muccioli G.G., Delzenne N.M. (2013). Cross-talk between Akkermansia muciniphila and intestinal epithelium controls diet-induced obesity. Proc. Natl. Acad. Sci. USA.

[58] Liu Z., DeSantis T.Z., Andersen G.L., Knight R. (2008). Accurate taxonomy assignments from 16S rRNA sequences produced by highly parallel pyrosequencers. Nucleic Acids Res..

[59] Claesson M.J., Wang Q., O’Sullivan O., Greene-Diniz R., Cole J.R., Ross R.P., O’Toole P.W. (2010). Comparison of two next-generation sequencing technologies for resolving highly complex microbiota composition using tandem variable 16S rRNA gene regions. Nucleic Acids Res..

