# Supplementary material for: Dietary Pea Fiber Supplementation Improves Glycemia and Induces Changes in the Composition of Gut Microbiota, Serum Short Chain Fatty Acid Profile and Expression of Mucins in Glucose Intolerant Rats
Source: Nutrients. 2017 Nov 12;9(11):1236. doi: 10.3390/nu9111236 (PMC5707708; doi:10.3390/nu9111236)
Supplement: Supplementary file 1 [file nutrients-09-01236-s001.docx]

**Supplementary Materials**

**Table S1.** Primer sequences for Real-time Polymerase Chain Reaction (RT-PCR).

| **Gene** | **Sequence (5’-3’)** | **Reference** |
| --- | --- | --- |
| **TLR2 (*tlr2***, **ID 310553)**  Forward  Reverse | GTACGCAGTGAGTGGTGCAAGT  GGCCGCGTCATTGTTCTC | [S1] |
| **TLR4 (*tlr4,* ID 29260)**  Forward  Reverse | AATCCCTGCATAGAGGTACTTCCTAAT  CTCAGATCTAGGTTCTTGGTTGAATAAG | [S1] |
| **Occludin (*ocln***, **ID 83497)**  Forward  Reverse | ATCTAGAGCCTGGAGCAACG  GTCAAGGCTCCCAAGACAAG | ___ |
| **ZO-1 (*tjp1*, ID 292994)**  Forward  Reverse | GCATGTAGACCCAGCAAAGG  GGTTTTGTCTCATCATTTCCTCA | ___ |
| **Muc1 (*muc1*, ID 24571)**  Forward  Reverse | TCGACAGGCAATGGCAGTAG  TCTGAGAGCCACCACTACCC | [S2] |
| **Muc2 (*muc2,* ID 24572)**  Forward  Reverse | GCACCTTCTTCAGCTGCATG  GCGCAGCCATTGTAGGAAAT | ___ |
| **Muc3 (*muc3*, ID 687030)**  Forward  Reverse | CTTGAGGAGGTGTGCAAGAAA  CCCCAGGGTGACATACTTTG | [S2] |
| **Muc4 (*muc4*, ID 303887)**  Forward  Reverse | GCTTGGACATTTGGTGATCC  GCCCGTTGAAGGTGTATTTG | [S2] |
| **GAPDH (*gapdh,* ID 24383)**  Forward  Reverse | GTGGCAGTGATGGCATGGAC  CAGCACCAGTGGATGCAGGG | ___ |
| ***Bifidobacterium* spp.**  Forward  Reverse | AGCAGTAGGGAATCTTCCA  CACCGCTACACATGGAG | [22] |
| **Total Bacteria**  Forward  Reverse | CGGYCCAGACTCCTACGGG  TTACCGCGGCTGCTGGCAC | [22] |

**Figure S1.** Schematic diagram showing the experimental outline of the study.

**Figure S2.** mRNA expression of toll-like receptors (TLRs) in adipose tissue and colon. Mean relative mRNA expression (FC, fold change) of (a) TLR2 in epididymal adipose tissue, (b, c) TLR2 and TLR4 in mucosal scrapings of colon normalized to glyceraldehyde-3-phosphate dehydrogenase (GAPDH) expression. Data are means ± SEM, n= 6-8. No significant differences were found between groups.

References

1. Le Mandat Schultz, A.; Bonnard, A.; Barreau, F.; Aigrain, Y.; et al. Expression of TLR-2, TLR-4, NOD2 and pNF-kappaB in a neonatal rat model of necrotizing enterocolitis*. PLoS One* **2007***, 2*, e1102.
2. Montoya, C.A.; Leterme, P.; Rome, V.; Beebe, S.; et al. Phaseolin from Phaseolus vulgaris bean modulates gut mucin flow and gene expression in rats*. Br. J. Nutr.* **2010**, 104, 1740-1747.
